# Supplementary figures and images for: Detecting temporal asymmetry after epilepsy surgery: a 3D MRI-based comparative outcome study of clinicians and lay observers
Source: PeerJ. 2025 Oct 30;13:e20201. doi: 10.7717/peerj.20201 (PMC12579851; doi:10.7717/peerj.20201)

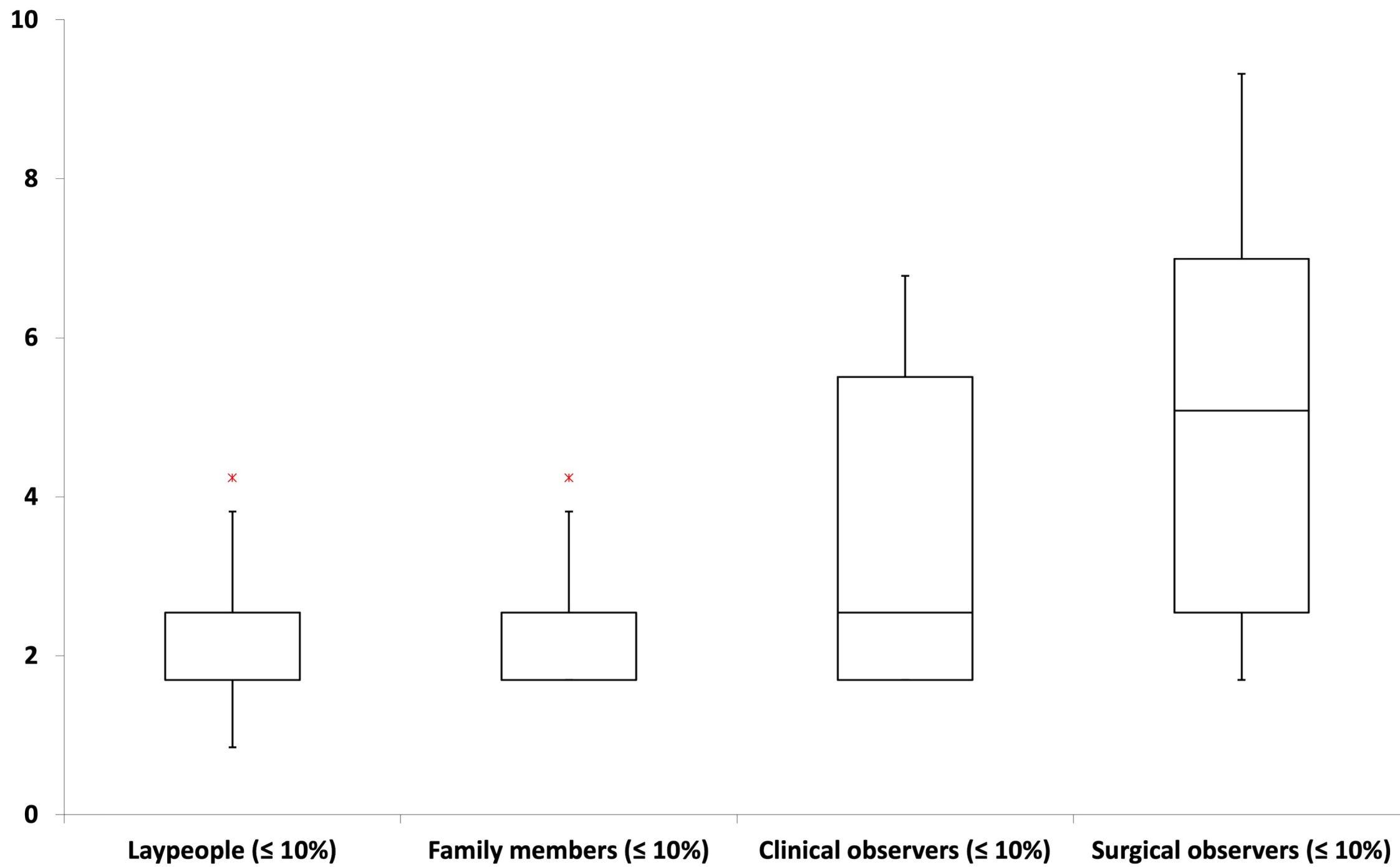

Supplement: Supplemental Information 3 — Box plots illustrating the distribution of detection rates of temporal asymmetry by individual observers based on three-dimensional craniofacial images with temporal thickness differences below 10%. For further details, refer to Table 6. [file peerj-13-20201-s003.pdf]

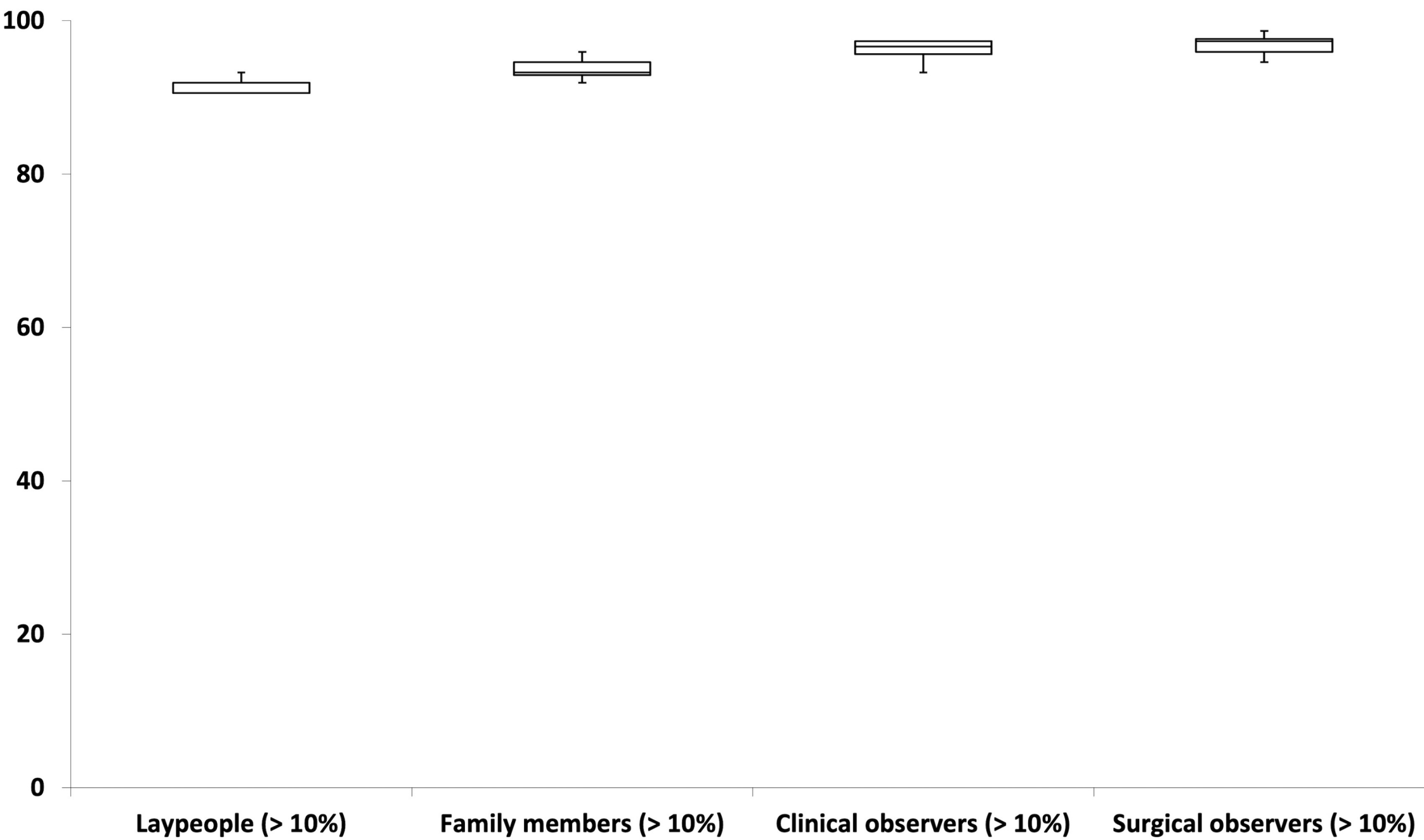

Supplement: Supplemental Information 4 — Box plots illustrating the distribution of detection rates of temporal asymmetry by individual observers based on three-dimensional craniofacial images with temporal thickness differences greater than 10%. For further details, refer to Table 6. [file peerj-13-20201-s004.pdf]
