# Supplementary material for: Detecting temporal asymmetry after epilepsy surgery: a 3D MRI-based comparative outcome study of clinicians and lay observers
Source: PeerJ. 2025 Oct 30;13:e20201. doi: 10.7717/peerj.20201 (PMC12579851; doi:10.7717/peerj.20201)
Supplement: Supplemental Information 5 [file peerj-13-20201-s005.docx]

**Table S1.** Reproducibility statistics for temporal thickness measurement.

| **Parameter** | **Intra-examiner** | | **Inter-examiner** | |
| --- | --- | --- | --- | --- |
|  | **Reliability**  **(ICC*)** | **Precision** | **Reliability**  **(ICC**)** | **Precision** |
|  |  | **REM** |  | **REM** |
| Mean | 0.99 | 1.06% | 0.94 | 5.00% |
| Min-Max | – | 0.08%–15.42% | – | 0.41%–16.24% |
| 95% CI | 0.98–0.99 | 0.04%–2.08% | 0.50–0.98 | 4.21%–5.80% |
| ICC, intra-class correlation, REM, relative error magnitude; Min, minimum; Max, maximum; CI, confidence interval.  *, absolute-agreement, 2-way mixed-effects model.  **, absolute-agreement, 2-way random-effects model.  ICC values: excellent reliability, >0.9; good, 0.75–0.9; moderate, 0.5–0.75; poor, <0.5.  REM scores: excellent, <1%; very good 1%–3.9%; good, 4%–6.9%; moderate 7%–9.9%; and poor >10%. | | | | |
