# Supplementary material for: Detecting temporal asymmetry after epilepsy surgery: a 3D MRI-based comparative outcome study of clinicians and lay observers
Source: PeerJ. 2025 Oct 30;13:e20201. doi: 10.7717/peerj.20201 (PMC12579851; doi:10.7717/peerj.20201)
Supplement: Supplemental Information 6 — Reliability statistics for panel assessment . [file peerj-13-20201-s006.docx]

**Table S2.** Reliability statistics for panel assessment.

| **Parameters** | **Reliability** | |
| --- | --- | --- |
|  | **Intra-observer**  **(**k**)** | **Inter-observer**  **(**k_Fleiss_**)** |
| **Untrained observers** (n=32) | 0.854±0.056 (0.834–0.874) | 0.842 (0.835–0.848) |
| Lay people (n=16) | 0.851±0.058 (0.820–0.881) | 0.844 (0.831–0.857) |
| Family members (n=16) | 0.857±0.056 (0.827–0.887) | 0.849 (0.836–0.862) |
| **Trained clinicians** (n=32) | 0.910±0.041 (0.895–0.925) | 0.876 (0.869–0.882) |
| Surgical observers (n=16) | 0.918±0.041 (0.896–0.939) | 0.876 (0.863–0.889) |
| Clinical observers (n=16) | 0.903±0.042 (0.881–0.925) | 0.891 (0.878–0.894) |
| **Overall** (n=64) | 0.882±0.057 (0.868–0.896) | 0.853 (0.850–0.856) |
| n, number of observers; k, Cohen’s Kappa; k_Fleiss_, Fleiss’s multirater kappa.  Intra-observer reliability reported as the mean ± standard deviation, with a 95% confidence interval.  Inter-observer reliability reported as the overall k_Fleiss_ value, with a 95% confidence interval.  Level of agreement: almost perfect, >0.90; strong, 0.80–0.90; and moderate, 0.60–0.79, and weak, <0.59 | | |
